# Identification and characterization of novel bat coronaviruses in Spain

**DOI:** 10.1371/journal.ppat.1013371

**Published:** 2025-08-04

**Authors:** Clàudia Soriano-Tordera, Jaime Buigues, Adrià Viñals, Elena Muscolino, Raquel Martínez-Recio, Juana Díez, Juan S. Monrós, José M. Cuevas, Jérémy Dufloo, Rafael Sanjuán

**Affiliations:** 1 Institute for Integrative Systems Biology (I^2^SysBio), Universitat de València and Consejo Superior de Investigaciones Científicas, València, Spain; 2 Institut Cavanilles de Biodiversitat i Biologia Evolutiva, Universitat de València, València, Spain; 3 Molecular Virology Group, Department of Medicine and Life Sciences, Universitat Pompeu Fabra, Barcelona, Spain; 4 Department of Genetics, Universitat de València, València, Spain; American University of Iraq Baghdad, IRAQ

## Abstract

The zoonotic transmission of bat coronaviruses poses a threat to human health. However, the diversity of bat-borne coronaviruses remains poorly characterized in many geographical areas. Here, we recovered eight coronavirus genomes by performing a metagenomic analysis of fecal samples from hundreds of individual bats captured in Spain, a country with high bat diversity. Three of these genomes corresponded to potentially novel coronavirus species belonging to the alphacoronavirus genus. Phylogenetic analyses revealed that some of these viruses are closely related to coronaviruses previously described in bats from other countries, suggesting a shared viral reservoir worldwide. Using viral pseudotypes, we investigated the receptor usage of the identified viruses and found that one of them can use human ACE2, albeit with lower affinity than SARS-CoV-2. However, the receptor usage of the other viruses remains unknown. This study broadens our understanding of coronavirus diversity and identifies research priorities for the prevention of zoonotic viral outbreaks.

## Author summary

Bats carry many different viruses, some of which can infect humans. Among these, bat coronaviruses are of particular concern. To be better prepared for future pandemics, it is important to understand how many of these viruses exist and their ability to infect different hosts. However, research in this area has often focused on certain parts of the world, while other regions remain underexplored. Spain has a rich diversity of bats, but very few studies have looked for coronaviruses in bats from the Iberian Peninsula. Here, we used viral metagenomics to test for the presence of coronaviruses in more than 200 bat samples collected across Spain. We identified eight coronavirus genomes, three of which may constitute new species. We also examined how closely related they are to previously known viruses, and whether they can use the same cellular receptors as known coronaviruses. Notably, we found that one of the viruses could use human ACE2, the SARS-CoV-2 receptor. Our findings reveal that bats in Spain host a diverse range of coronaviruses, including some that could potentially infect humans. This highlights the importance of studying coronavirus diversity more broadly worldwide.

## Introduction

Bats are taxonomically diverse mammals that represent 20% of all mammal species [1,2]. Bats have been identified as natural reservoirs for numerous zoonotic viruses, including the Nipah and Hendra paramyxoviruses [3], the hemorrhagic Ebola filovirus [4], or the severe acute respiratory syndrome (SARS) and Middle East respiratory syndrome (MERS) coronaviruses [5–7]. Their frequent association with viral emergence might simply reflect the diversity and abundance of bat species [8]. Alternatively, it has been suggested that bats exhibit increased propensity to carry and transmit pathogens because of their distinctive metabolism associated with flight, limited immunoinflammatory responses, tendency to aggregate into populous colonies, and adaptation to peri-urban habitats [1,2,9]. Given their role as viral reservoirs, there has been a concerted research effort to explore viral diversity and identify potential zoonotic viruses in bats [10–13]. This has been facilitated by the development of metagenomic next-generation sequencing (mNGS) that allows exploring the unknown virosphere and enables the identification of many new viral species, including novel bat-borne viruses [14,15].

Numerous studies have suggested that bats are frequent carriers of coronaviruses [16]. Coronaviruses are enveloped, single-stranded, positive-sense RNA viruses with genomes ranging from 26 to 31 kb. The family *Coronaviridae* is divided into four genera: *Alphacoronavirus* (AlphaCoV) and *Betacoronavirus* (BetaCoV), which are associated with infections in mammals, and *Gammacoronavirus* and *Deltacoronavirus* that primarily infect birds but also occasionally mammals [17,18]. To date, seven coronaviruses have successfully jumped from animal reservoirs to humans (SARS-CoV, SARS-CoV-2, MERS-CoV, 229E, NL63, HKU1 and OC43), and additional coronaviruses have been detected during background surveillance as causative agents of isolated human infections [19,20]. Assessing the zoonotic potential of coronaviruses identified in wildlife is therefore critical, but not an easy task. Indeed, many deposited sequences are only partial, the full virus is often not isolated, and working with unknown pathogens requires stringent biosafety measures. One way to overcome these limitations is to use surrogate experimental systems that focus on specific steps of the viral life cycle, such as viral entry, which is mediated by the coronavirus spike glycoprotein and plays a critical role in determining viral host range and cellular tropism [21]. Viral pseudotypes, in which the spike glycoprotein is incorporated into a viral vector, can be used to safely and faithfully characterize the receptor usage of a new virus and its ability to enter human cells [13,22,23].

Given the threat they pose to human health, identifying and characterizing bat-borne coronaviruses is a priority worldwide. However, there is a strong geographical bias in the metagenomic identification of bat coronaviruses. As of November 2024, 60.4% of the coronavirus sequences deposited in the Bat-associated virus database DBatVir [24] originated from Asia, while only 6.5% have been detected in European bats. Moreover, only a few studies have been carried out to search for coronaviruses in Iberian bats and none of them provided a direct *in vitro* assessment of the zoonotic potential of the identified viruses [25,26], despite the Iberian Peninsula being home to a wide diversity of bat species. Here, we used mNGS to detect coronaviruses in a large number of fecal samples from different regions of Spain. Eight coronavirus genomes were recovered, including three potential novel species.

## Results

### Coronaviruses found in bat fecal viromes

**Fig 1:**
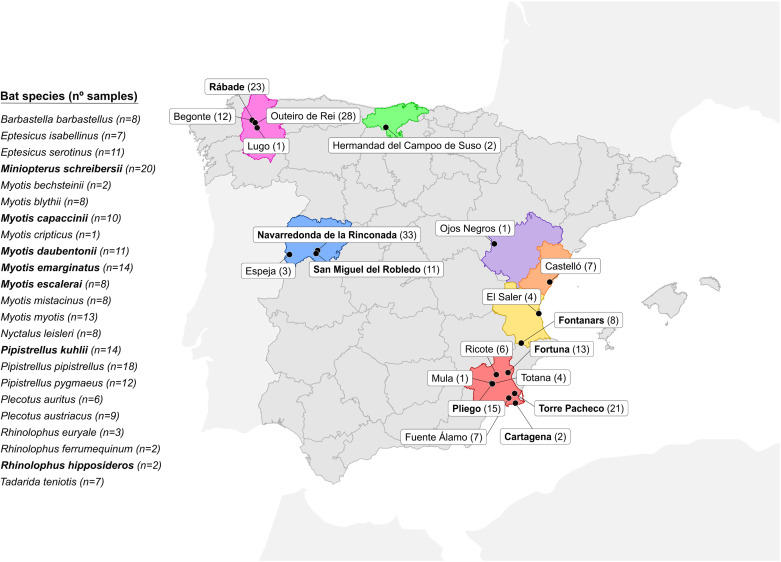
Collection of fecal samples from 23 bat species across Spain. The number of samples collected from each bat species and the number of individuals captured in each area are indicated in parentheses. The locations and bat species in which coronaviruses were detected are shown in bold. The provinces where samples were collected are coloured in red (Murcia), yellow (Valencia), orange (Castellón), purple (Teruel), blue (Salamanca), pink (Lugo) and green (Cantabria). This map was created using R (https://www.R-project.org/) and the geospatial data of Spain obtained from GADM v4.1 (https://geodata.ucdavis.edu/gadm/gadm4.1/shp/gadm41_ESP_shp.zip). GADM data are freely available for academic use (https://gadm.org/license.html).

We obtained 202 fecal samples from 23 bat species in 20 collection points across Spain (Fig 1). Samples were grouped into 26 pools, each containing samples from individuals assigned to the same bat species according to morphological trait visualization (S1 Table). After RNA extraction and library preparation, Illumina sequencing yielded between 4.5 and 48 million raw reads per pool (S2 Table). Quality-filtered reads were assembled *de novo*, and the resulting contigs were used to identify viral sequences. A total of 8946 viral contigs and 430 high-quality complete or nearly complete viral genomes were obtained, the vast majority of which corresponded to bacteriophages. In this study, we focused on reads corresponding to the family *Coronaviridae*, while other viral families were analyzed in previous articles [27–29] (S2 Table). We directly assembled six contigs corresponding to coronavirus sequences, as determined by CheckV [30]. The number of reads assigned to each virus ranging from 266 (mean coverage 12.44) to 47,005 (mean coverage 223.73; S3 Table). We additionally obtained 155 coronavirus contigs corresponding to small genome regions (S2 Table). For each of these contigs, we searched the closest complete or quasi-complete database sequence and used it to remap all reads. However, the resulting sequences were highly fragmented, incomplete (>90% unknown nucleotides), and with low coverage. All except two such sequences were hence discarded. Of the two partial genomes retained, one was successfully completed by PCR amplification of the missing regions using specific primers (S4 Table). We therefore finally obtained five complete, two quasi-complete genomes and one partial genome. The proposed names of each virus and sequence accession numbers are presented in Table 1.

**Table 1:** Features of *de novo* assembled coronavirus sequences.

Accession number	Proposed names	Abbreviated names	Bat species	Length (nt)	Genus	Subgenus
PQ611137	Sarbecovirus sp. isolate RhBetaCoV_Murcia2022	RhBetaCoV-Murcia2022	*Rhinolopus hipposideros*	29259	Betacoronavirus	Sarbecovirus
PQ611138	Alphacoronavirus sp. isolate McAlphaCoV_Yeseras	McAlphaCoV-Yeseras	*Myotis capacinii*	28323	Alphacoronavirus	Pedacovirus
PQ611139	Alphacoronavirus sp. isolate MeAlphaCoV_Sima	MeAlphaCoV-Sima	*Myotis escalerai, Myotis emarginatus*	24428	Alphacoronavirus	Pedacovirus
PQ611140	Alphacoronavirus sp. isolate MsAlphaCoV_Gordo	MsAlphaCoV-Gordo	*Miniopterus schreibersii*	29264	Alphacoronavirus	Minunacovirus
PQ611141	Alphacoronavirus sp. isolate MsAlphaCoV_Murcia2022	MsAlphaCoV-Murcia2022	*Miniopterus schreibersii*	28877	Alphacoronavirus	Minunacovirus
PQ611142	Alphacoronavirus sp. isolate PkAlphaCoV_Valencia2022	PkAlphaCoV-Valencia2022	*Pipistrellus kuhlii*	28171	Alphacoronavirus	Nyctacovirus
PQ738185	Bat coronavirus isolate BtCoV/13585–58/M.dau/DK/2014 isolate MdAlphaCoV-Spain2022	MdAlphaCoV-Spain2022	*Myotis daubentonii*	27302*	Alphacoronavirus	Pedacovirus
PQ738186	Bat coronavirus HKU7 isolate MsAlphaCoV-Spain2022	MsAlphaCoV-Spain2022	*Miniopterus schreibersii*	2953**	Alphacoronavirus	Minunacovirus

*Reconstructed by remapping and PCR amplification.

**Incomplete viral genome.

### Phylogenetic and taxonomic classification of coronavirus sequences

**Fig 2:**
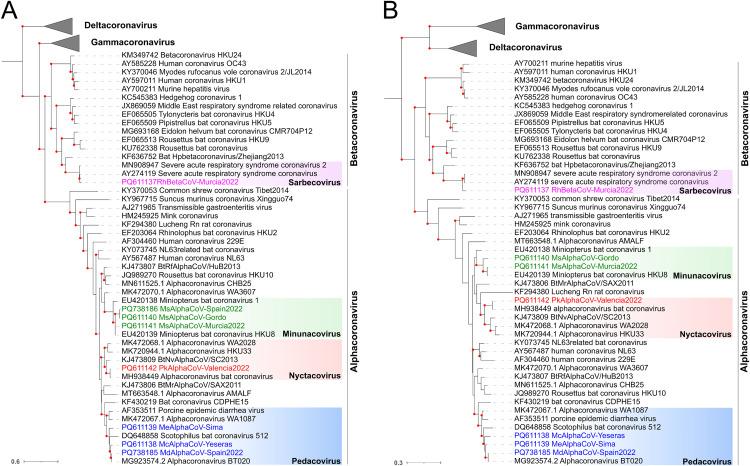
Phylogenetic positioning of the newly described viruses within the family *Coronaviridae.* Maximum-likelihood (ML) trees were built using RdRP (A) and helicase (B) amino acid sequences. Sequences from representative ICTV-approved viral species were included. The Gamma- and Deltacoronavirus taxonomic groups are collapsed by genus. Microhyla letovirus 1 was used as an outgroup to root the trees. Viruses described in this study are shown in colors. Bootstrap values higher than 80 are indicated with red circles. The scale bar indicates the evolutionary distance in amino acid substitutions per site.

To determine the genus and subgenus classification of the identified coronaviruses, we constructed phylogenetic trees using the RNA-dependent RNA polymerase (RdRP; Fig 2A), and helicase (Fig 2B) amino acid sequences of ICTV-approved species [18]. One sequence (MsAlphaCoV-Spain2022) could not be included in the helicase tree due to the lack of this region in the partial genome. These analyses demonstrated that seven of the eight coronaviruses were alphacoronaviruses, including three minunacoviruses (MsAlphaCoV-Gordo, MsAlphaCoV-Murcia2022 and MsAlphaCoV-Spain2022), one nyctacovirus (PkAlphaCoV-Valencia2022), and three pedacoviruses (MsAlphaCoV-Yeseras, MeAlphaCoV-Sima and MsAlphaCoV-Spain2022), whereas one was a betacoronavirus belonging to the *Sarbecovirus* subgenus (RhBetaCoV-Murcia2022).To ascertain whether these could be novel species, we determined the percent sequence identity to the closest nucleotide sequences using BLASTn. Five of the sequences showed >91% nucleotide identity to previously described viruses, while three (McAlphaCoV-Yeseras, MeAlphaCoV-Sima and MsAlphaCoV-Gordo) showed <84% nucleotide identity to any other known coronaviruses and thus might represent new species (S3 Table).

### Geographical distribution and prevalence of identified coronaviruses

To determine geographical distribution and possible prevalence of these viruses, we used RT-PCR to detect their presence in the individual fecal samples from the pools in which they were initially identified (S5 Table) using specific primers (S6 Table). We found that several viruses were present in multiple individuals from the same location, suggesting intra-roost virus circulation. Notably, PkAlphaCoV-Valencia2022 was detected in half (4/8) of the *Pipistrellus kuhlii* samples from in the Fontanars municipality in the province of Valencia, whereas it was absent from 6 samples obtained in other regions of Spain. Similarly, MeAlphaCoV-Sima was present in 2/3 samples from the Pliego municipality in the province of Murcia, but again absent from 6 additional samples from other regions. Viruses were only detected in a single sampling spot, except for McAlphaCoV-Yeseras, which was found in samples from two different municipalities in the province of Murcia (Fortuna and Pliego, with 3/5 and 1/4 positives, respectively), suggesting that this virus may be circulating in two separate bat roosts. Among the others, MdAlphaCoV-Spain2022 was found in 2/9 samples from the Rábade municipality (Lugo province), whereas MsAlphaCoV-Gordo was detected in 2/9 individual samples from Torre Pacheco municipality (Murcia). The other two viruses, RhBetaCoV-Murcia2022 and MsAlphaCoV-Murcia2022, were present in only one sample each from Cartagena (Murcia) and Torre Pacheco (Murcia), respectively. Overall, most positive samples came from the province of Murcia, which was the most extensively sampled area and where bats are known to exhibit particularly high abundance and diversity, with at least 20 of the 35 species present in Spanish territory found in this province [31]. Finally, each of the 17 positive individual samples was used to check bat species identity by cytochrome B sequencing (S7 Table). All identifications based on visualization of morphological traits were confirmed, except one of the two MeAlphaCoV-Sima-positive samples from Pliego (Murcia), which was identified by sequencing as *Myotis emarginatus* instead of *M. escalerai*, suggesting transmission of the virus between these two closely related and morphologically similar hosts.

### Analysis of the closest spike sequences

**Fig 3:**
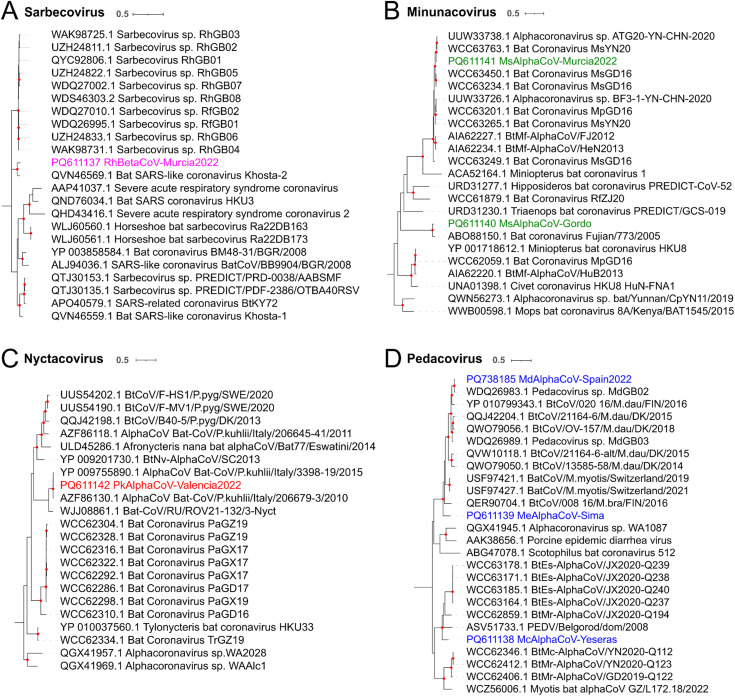
Spike sequences in the context of *Sarbecovirus (A)*, *Minunacovirus (B)*, *Nyctacovirus (C)* and *Pedacovirus (D)* phylogenies. ML trees were built using the 20 BLASTp hits for each identified spike sequence and the ICTV-approved viral species for each subgenus. Viruses described in this study are shown in colors. Bootstrap values higher than 80 are indicated with red circles. The scale bar indicates the evolutionary distance in amino acid substitutions per site.

To further investigate the evolutionary origin of the detected coronaviruses, spike phylogenetic trees were constructed for each subgenus using the 20 closest BLASTp hits for each sequence. ICTV-approved viral species from each subgenus were added to the analysis in case they were not already present among the 20 hits. This analysis was done only for the seven complete or almost complete sequenced viruses, since the other sequence lacked spike reads. We found that the RhBetaCoV-Murcia2022 spike was related to another sarbecovirus, the bat SARS-like Khosta-2 (QVN46569.1) found in *Rhinolophus hipposideros* bats from Russia [32] (93% amino acid identity; Fig 3A and S3 Table), while both were relatively distant to human SARS coronaviruses. The spike of MsAlphaCoV-Gordo was related with 92% amino acid identity to another minunacovirus found in *Miniopterus schreibersii* in China (ABO88150.1) [33] (Fig 3B and S3 Table), while the spike of the other minunacovirus, MsAlphaCoV-Murcia2022, was most closely related to a virus detected in *Rhinolophus sinicus* (UUW33738.1; 97% amino acid identity) also in China [34] (Fig 3B and S3 Table). The spike of the nyctacovirus identified in *Pipistrellus kuhlii* (PkAlphaCoV-Valencia2022) was highly similar (up to 98% amino acid identity) to the spike of viruses found in the same bat species in Italy (YP009755890.1 and AZF86130.1) [35] (Fig 3C and S3 Table). The spike of the pedacovirus from *Myotis capacinii* (McAlphaCoV-Yeseras) formed a clade with those of viruses found in China (WCC63178.1, WCC63185.1, WCC63171.1, WCC63164.1 and WCC62859.1) [36] and in Russia (ASV51733.1) [37], but shared less than 78% amino acid identity with any of these sequences (Fig 3D and S3 Table). For the other pedacovirus spike, MeAlphaCoV-Sima, the closest spike sequence was obtained from one virus detected in *Myotis myotis* in Switzerland (USF97421.1) [38] (80% amino acid identity) (Fig 3D and S3 Table). Finally, the pedacovirus identified in *Myotis daubentonii* (MdAlphaCoV-Spain2022) was highly similar to a virus identified in the same bat species in United Kingdom (WDQ26983) [13] (93% amino acid identity).

### Failure to isolate the newly identified coronaviruses

To functionally characterize these newly identified viruses, we attempted to isolate the three novel alphacoronaviruses (McAlphaCoV-Yeseras, MeAlphaCoV-Sima and MsAlphaCoV-Gordo) and the sarbecovirus (RhBetaCoV-Murcia2022) from PCR-positive fecal samples. Briefly, Huh-7 and VeroE6 cells were inoculated with positive samples in the presence or absence of trypsin, and viral load was followed by RT-qPCR using specific primers (S8 Table). However, after four days, no significant viral replication could be measured. A second passage was attempted for the McAlphaCoV-Yeseras and the RhBetaCoV-Murcia2022 but was also unsuccessful.

### RhBetaCoV-Murcia2022 can use human and bat ACE2 to enter cells

**Fig 4:**
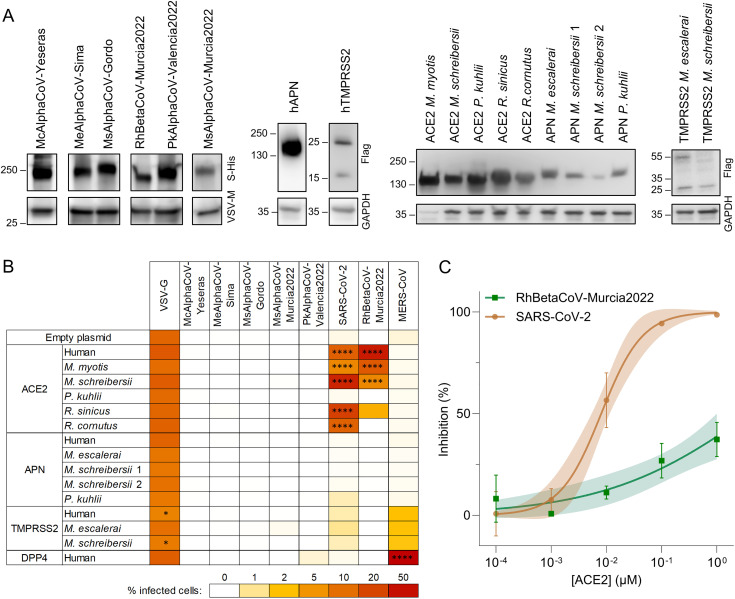
Receptor usage of the identified coronaviruses. A. Western blot validation of spike incorporation into VSV pseudotypes and receptor expression. The spike was detected with an anti-His-Tag antibody and VSV-M was used as a loading control, whereas receptors were detected with an anti-Flag-Tag antibody and GAPDH was used as a loading control. B. Heat map showing the percentage of infected cells after transfection of the indicated bat or human orthologues and inoculation with pseudotyped viruses carrying the indicated spike proteins. For each combination, the average of three replicate (n = 3) assays are shown. The two APN alleles present in *M. schreibersii* were considered. A two-way ANOVA with Dunnett’s correction for multiple tests was performed to compare all the receptors to the empty. * P < 0.05, **** P < 0.0001. C. Infection inhibition assay by soluble hACE2 against RhBetaCoV-Murcia2022 and SARS-CoV-2 VSV pseudotypes. Each dot is the average of two technical replicates, lines correspond to a sigmoidal 2-parameter fit, and shaded areas correspond to 95% confidence intervals.

In the absence of viral isolates, we used vesicular stomatitis virus (VSV)-based pseudotyping to explore the receptor usage. We successfully constructed six viral pseudotypes, as shown by detection of spike incorporation into viral particles by Western blot (Fig 4A). We then tested whether these pseudotypes could enter cells overexpressing the human (h) orthologues of the known coronavirus receptors ACE2, APN, DPP4 and TMPRRS2. The expression of each receptor was also verified by Western blot (Fig 4A), except for hACE2 and hDPP4, whose expression was confirmed by infection with SARS-CoV-2 and MERS-CoV pseudotypes, respectively (Fig 4B). Interestingly, RhBetaCoV-Murcia2022 pseudotypes efficiently entered hACE2-expressing cells (Fig 4B), consistent with previous results obtained with the related Khosta-2 spike [39]. The other five viruses could not enter cells expressing any of the four human receptors tested. However, given that interspecies variation in receptor proteins can strongly affect the ability of a virus to use them for entry [39,40], we sought to determine whether the identified spikes could use various bat ACE2, APN or TMPRSS2 orthologues. Unfortunately, none of these bat genomes have been sequenced, except for *Pipistrellus kuhlii*. To address this limitation, we managed to obtain novel mRNA sequences from bat feces samples for ACE2 from *Miniopterus schreibersii*, APN from *Myotis escalerai* and *Miniopterus schreibersii* and TMPRSS2 from *Myotis escalerai* and *Miniopterus schreibersii.* For the other gene-bat species combinations, RT-PCR from fecal samples was unsuccessful and we thus resorted on available sequences from the related *Myotis myotis*, *Rhinolophus sinicus* and *Rhinolophus cornutus* species. Sequences were synthesized and correctly expressed, as shown by Western blot (Fig 4A). Moreover, as a functional control, we verified that the ACE2 orthologues of *Myotis myotis*, *Miniopterus schreibersii*, *Rhinolophus sinicus* and *Rhinolophus cornutus* allowed SARS-CoV-2 spike-mediated entry, as previously shown [41] (Fig 4B). We found that, in addition to using hACE2, the RhBetaCoV-Murcia2022 pseudotype could enter cells overexpressing the ACE2 orthologues from *Myotis myotis*, *Miniopterus schreibersii* and *Rhinolophus sinicus* (Fig 4B). However, the other five viruses could not use any ACE2, APN or TMPRSS2 orthologue tested. Therefore, the receptor usage of these viruses remains unclear.

### The RhBetaCoV-Murcia2022 spike shows weaker hACE2 binding than the SARS-CoV-2 spike

Since the RhBetaCoV-Murcia2022 spike can use hACE2 for entry, we set out to compare its receptor binding affinity with that of the SARS-CoV-2 spike. For this, we carried out competition assays in which VSV pseudotypes bearing the SARS-CoV-2 or RhBetaCoV-Murcia2022 spike were preincubated with soluble hACE2 and then used to inoculate hACE2-expressing HEK cells (Fig 4C). We found that hACE2 blocked entry of the SARS-CoV-2 pseudotype more efficiently (IC50 = 0.0080 ± 0.0034 µM) than for RhBetaCoV-Murcia2022 (IC50 > 1 µM), indicating low hACE2 binding affinity for the RhBetaCoV-Murcia2022 spike relative to SARS-CoV-2. Therefore, this virus appears to be poorly adapted to using the human ACE2 orthologue.

## Discussion

We have characterized the feces virome of 23 different bat species from various regions of Spain and identified five complete, two quasi-complete and one incomplete coronavirus genomes, including three candidate novel species. Of the eight identified viruses, five exhibited close genetic similarity to previously described coronaviruses identified in the same or different bat species from distant geographic regions, particularly in Asia and other parts of Europe. For instance, variants of RhBetaCoV-Murcia2022 were detected in the same bat species (*Rhinolophus hipposideros*) in Russia [32] and in the United Kingdom, as well as MsAlphaCoV-Murcia2022 isolated in *Miniopterus schreibersii* was previously obtained from another bat species (*Rhinolophus sinicus*) in China [34]. This suggests a broad geographical distribution of some bat-borne coronaviruses, potentially facilitated by bat migratory patterns and habitat overlap across the continent. Moreover, the fact that bats from different families harbour variants of the same viral species highlights the broad species tropism of some bat-borne coronaviruses. Such inter-family or inter-genus cross-species transmission of alpha- and betacoronaviruses was shown to occur frequently in Asia and America [42,43] and our data suggest that this may also be the case in Europe. Finally, the identification of three novel bat-borne coronavirus species expands our knowledge of coronavirus diversity in Europe, particularly in Spain, where studies searching for bat-borne coronaviruses have been limited [25,26].

Although metagenomic studies allow an in-depth characterization of the unknown virosphere in wildlife, experimental characterization of newly identified viruses is critical to assess their zoonotic potential. Given the presumed importance of spike-mediated entry in coronavirus cross-species transmission, we used spike-expressing viral pseudotypes to assess the receptor usage of the six coronaviruses initially identified. We showed that RhBetaCoV-Murcia2022 can use hACE2 as a receptor. Although this raises concerns about its potential zoonotic risk, this does not imply that the virus is currently capable of infecting humans. Indeed, additional incompatibilities with host proteases, immune response, or post-entry blocks in viral replication may prevent RhBetaCoV-Murcia2022 from replicating in human cells [1,16]. Moreover, this spike showed much weaker binding to the hACE2 receptor than SARS-CoV-2. The critical amino acids for hACE2 binding are located in the receptor binding motif of the spike protein [44], a highly variable region where multiple changes occur among SARS-related viruses. RhBetaCoV-Murcia2022 spike is very similar to that of Khosta-2, for which it has been described that these key residues are widely different from those in SARS-CoV-2 and SARS-related viruses [32]. Similarly, other viruses distantly related to SARS-CoV-2 were recently found to bind ACE2, albeit with very low affinity [45]. Finally, to better examine their zoonotic potential, we tried to isolate the three new alphacoronavirus (McAlphaCoV-Yeseras, MeAlphaCoV-Sima and MsAlphaCoV-Gordo) and the RhBetaCoV-Murcia2022 betacoronavirus from PCR-positive samples, but this was unsuccessful. This could be due to low viral loads in samples, the absence of infectious virus despite the presence of viral RNA, the choice of non-susceptible cell lines for isolation, or a suboptimal dose of trypsin used, among other potential causes. Therefore, further experiments are needed to assess the ability of these viruses to replicate in human cells.

Most of the coronaviruses we identified could not use any of the tested bat orthologues of ACE2, APN or TMPRSS2. The only exception was the RhBetaCoV-Murcia2022, which could use the ACE2 orthologues of *Myotis myotis*, *Miniopterus schreibersii* and *Rhinolophus sinicus*, but not from *Rhinolophus cornutus,* despite a 95.3% protein identity between these last two species. Previous studies have demonstrated that minimal interspecies sequence variations among receptor proteins can deeply alter their ability to mediate viral entry [39,40]. A limitation of our study is that we were not always able to test the receptor orthologues of the actual bat species where the viruses were identified. The genomes of some bat species are not available and attempts of gene amplification from feces samples were often unsuccessful. Research efforts such as the Bat1K project (https://bat1k.com), which aims to sequence the genomes of all 1400 living bat species, will help to fill this knowledge gap and will allow to precisely assess the ability of a virus to use receptor orthologues of the bat species where it was identified.

Therefore, although we cannot completely exclude that the identified viruses may use ACE2, APN, DPP4 or TMPRSS2 from their host species as a receptor, it is likely that they use other yet unknown receptors for entry. Indeed, three of the identified viruses were minunacoviruses and one was a nyctacovirus, and no receptor has been described yet for viruses of these subgenera. Finally, three of the identified alphacoronaviruses belonged to the pedacovirus subgenus, named after the porcine epidemic diarrhea virus (PEDV). Although PEDV has been suggested to use APN as a receptor [46,47], this has been debated recently as no specific interaction between the PEDV spike and APN could be measured [48,49] and APN knockout pigs are susceptible to PEDV infection [50]. Therefore, PEDV and other bat-borne pedacoviruses may use a receptor other than APN.

In conclusion, our results highlight the role of bats as a global reservoir of a wide range of coronaviruses, including Spain. Future work aimed at characterizing the diversity of coronaviruses in different host types and regions, as well as ecological monitoring of interactions between bats and other species, including humans, will be crucial to mitigate the risks of future pandemics.

## Materials and methods

### Ethics statement

Samples consisted of feces from wild animals captured using nylon mist nets or a harp trap. Bats were kept briefly in cotton bags until fresh fecal samples were obtained. According to the European Directive regulating the protection of animals used for scientific purposes (2010/62/EU, Article 1), subsequently transposed into Spanish legislation (Royal Decree 53/213, 1 February, Article 2), procedures used in this study are not subject to the condition of animal experimentation and therefore do not require approval by an institutional ethics committee, but specifically a permit for fieldwork from the competent regional authority. The necessary permits from the Generalitat Valenciana for the sampling of wild bats were granted under Exp. 2022-VS (FAU22_009).

### Sample collection

To capture bats from their natural habitats, nylon mist nets (Ecotone) and harp traps (Austbat) were used in seven Spanish regions (Cantabria, Castellón, Lugo, Murcia, Salamanca, Teruel, and Valencia) between May and October 2022 (S1 Table **and Fig 1**). Each captured animal was identified at the species level using morphological keys, sexed, measured, aged, and briefly placed in cotton bags to recover fresh fecal samples. Samples were obtained from 202 individuals representing 23 species of bats (19 from the *Vespertilionidae* family, 3 from the *Rhinolophidae* family and 1 from the *Molossidae* family). Exceptionally, fresh samples from European free-tailed bat (*Tadarida teniotis*) were collected from a colony without trapping involved. Fecal samples were placed in tubes containing 500 µL of phosphate-buffered saline (PBS) and maintained at 4ºC throughout the duration of the fieldwork (6–9 h). Samples were then kept at -20°C until arrival at the laboratory where they were stored at -80°C.

### Sample processing and RNA extraction

A fraction of the fecal samples from each of the 202 individuals was combined into 26 pools, each one containing between 1 and 15 samples from the same bat species as initially determined using morphological traits (S1 Table). Prior to sample processing, each pool was spiked with 10^5^ plaque-forming units (PFU) of VSV as a positive control to assess the final viral recovery efficiency. Each tube was mixed with PBS, resulting in a final volume of 1.5 mL. Fecal samples were homogenized using the Precellys Evolution tissue homogenizer (Bertin) in 2 mL tubes with 1.4 mm ceramic beads (Precellys), using three cycles of 30 sec at 6500 rpm, with 10 sec pause between cycles. The homogenates were centrifuged twice at 20000 g for 3 min at 4°C. The resulting supernatants were filtered using a Minisart cellulose acetate syringe filter with a 1.2-µm pore size (Sartorius) and transferred to ultra-clean 2 mL tubes (Eppendorf). RNA extraction was performed using 280 µL of the total filtered volume with the QIAamp Viral RNA minikit (Qiagen). RNA was eluted in a final volume of 40 µL and stored at -80°C.

### Sequencing and annotation

The extracted RNA was subjected to library preparation using the stranded mRNA preparation kit (Illumina) but starting at the fragmentation stage. Samples were subjected to paired-end sequencing using a NextSeq 550 device with a read length of 150 bp at each end (S2 Table). Raw reads were deduplicated, quality filtered with a quality trimming threshold of 20, and any reads below 70 nucleotides in length were removed using fastp v0.23.2 [51]. *De novo* sequence assembly was performed using SPAdes v3.15.4 with the meta option [52], as well as using MEGAHIT v1.2.9 [53] with default parameters. Assembled contigs were clustered to remove replicates or small replicates of larger contigs, using CD-HIT v4.8.1 [54]. Contigs shorter than 1000 nt were removed and the remaining sequences were taxonomically classified using Kaiju v1.9.0 [55] with the subset of the National Center for Biotechnology Information (NCBI) nr protein database comprising archaea, bacteria and viruses (downloaded on June 6, 2023). Then, all clustered sequences were analyzed using Virsorter2 v2.2.4 [56] to detect viral contigs. Following this, the quality of the viral contigs was further assessed using CheckV v1.0.1 with the CheckV database v1.5. Contigs corresponding to phages and those that could not be classified into a known viral family were excluded. The remaining contigs were selected based on their size, completeness, and the ability of the assigned virus family to infect vertebrates. Finally, contigs that were assigned to the *Coronaviridae* family were selected. Coverage statistics were obtained by remapping the trimmed and filtered reads to each contig using Bowtie2 v2.2.5 [57] (S3 Table). Open reading frames (ORFs) were predicted using ORFfinder (ncbi.nlm.nih.gov/orffinder), while protein domains were annotated using InterProScan v5.63-95.0 [58] with the Pfam database v35.0.

### Reconstruction of partial genomes

For contigs corresponding to partial coronavirus genomes, a BLASTn was performed to identify the closest complete or quasi-complete (>20 kb) sequences available in GenBank. Then, our trimmed and filtered reads were remapped to these references using Bowtie2 v2.2.5 [57] and the bam file generated was used to obtain a consensus sequence with iVar v1.4.4 [59] adjusting the minimum frequency threshold (t = 0.8), the minimum depth to call consensus (m = 6), and the minimum insertion frequency threshold (c = 0.5). However, since the resulting sequences contained missing regions larger than 8 kb, no additional attempts were made to recover full genomes from these sequences. In one case, however, gaps were sufficiently small (<3.5 kb) to carry out PCR amplification of the missing regions. For this, we designed specific primers annealing at the end of the known sequence regions (S4 Table). PCR products were obtained using the Phusion Plus DNA polymerase (Thermo Scientific), purified using the DNA Clean & Concentrator-5 kit (Zymo Research) and sequenced by Sanger with the same primers used for the amplification.

### Phylogenetic and taxonomical positioning

To place the sequences within the global coronavirus diversity, the *Coronaviridae* ICTV report [18] was used to identify representative species. RdRP and helicase amino acid sequences from representative coronavirus species were downloaded from the NCBI. The sequence of Microhyla letovirus 1 was included as an outgroup for comparative purposes. Sequences were aligned with Clustal Omega v1.2.3 [60] and a maximum likelihood (ML) tree was constructed under the LG + I + G4 substitution model. Phylogenetic analyses were conducted using IQTree v2.0.3 [61], and model selection was performed with the built-in ModelFinder feature [62]. Branch support was estimated using ultra-fast boot-strapping replicates (UFBoot2) [63] and an approximate likelihood-ratio test (SH-aLRT) [64] with 1000 replicates. The resulting phylogenetic trees were visualized using iTol v6.0.9 [65]. To assess whether the identified viruses represented novel species, the percentage of nucleotide sequence identity was obtained for the closest viral genomes using BLASTn (S3 Table).

### RT-PCR amplification of viral sequences

RT-PCR was used to check for the presence of the identified coronaviruses in each of the individual samples within each positive pool (S5 Table). RNA was extracted from each individual fecal sample using the Qiagen QIAamp Viral RNA minikit (Qiagen), following manufacturer’s instructions, and eluted in a final volume of 30 µL. Specific primers were designed for cDNA synthesis and subsequent PCR amplification of a 500–800 bp spike region of each virus of interest (S6 Table). A volume of 4 µL of the RNA extraction was used for cDNA synthesis using Invitrogen’s Superscript IV enzyme (Invitrogen). For PCR, the NZYTaq II Green Master Mix (NZYTech) was used. Sample positivity was checked by electrophoresis on a 1% agarose gel using NZYTech Green Safe Premium (NZYTech).

### Bat taxonomic confirmation of the positive coronavirus samples

Bats were initially classified by visualization of morphological traits during feces acquisition. To confirm this, we determined cytochrome B gene sequences for each of the 17 positive individual feces samples (S7 Table). For this, we extracted nucleic acids using the same protocol as described above, since the QIAamp Viral RNA minikit (Qiagen) is also routinely used for DNA co-purification [28,66]. We next amplified by PCR and Sanger sequenced a 148 bp region of the cytochrome B gene using specific primers (F_L15601_VirCitB: TACGCAATCCTACGATCAATTCC; and R_H15748_VirCitB: GGTTGTCCTCCAATTCATGTTAG) as described previously [67]. The closest GenBank nucleotide sequences were searched using BLASTn.

### Phylogenetic analysis of spike protein sequences

To explore the evolutionary origin of the identified coronaviruses, we constructed spike phylogenetic trees of each subgenus using the closest amino acid sequences from each identified sequence selected with BLASTp and all ICTV-approved species for each subgenus (S3 Table). Spike sequences were downloaded from the NCBI. The sequence of Microhyla letovirus 1 was included as an outgroup for comparative purposes. Sequences were aligned with Clustal Omega v1.2.3 [60] and a ML tree was constructed under the WAG + F + G4 substitution model. Phylogenetic analyses were conducted as previously described.

### Cell culture

HEK293T cells were obtained from the American Type Culture Collection (ATCC, CRL-3216) and cultured in Dulbecco’s Modified Eagle’s Medium (DMEM; Gibco) supplemented with 10% fetal bovine serum (FBS, Gibco), 1% non-essential amino acids (NEAA; Gibco), penicillin and streptomycin (P/S; 10 units/mL and 10 µg/mL, respectively; Gibco) and amphotericin B (250 ng/mL, Gibco). Huh7 cells were kindly provided by Francis Chisari and VeroE6 cells were obtained from the ATCC (ATCC-1586). Both were maintained in DMEM supplemented with 10% FBS (Sigma), 1% P/S (Gibco) and 1% NEAA (Gibco). All cells were maintained at 5% CO_2_ and 37°C in a humidified incubator and were routinely screened for the presence of mycoplasma by PCR.

### Viral isolation attempts

Fecal samples positive for the presence of the novel alphacoronaviruses (McAlphaCoV-Yeseras, MeAlphaCoV-Sima and MsAlphaCoV-Gordo) and the betacoronavirus (RhBetaCoV-Murcia2022) were used to inoculate VeroE6 and Huh-7 cells. Cells were seeded in 12-well plates to achieve an 80% confluence on the day of infection and incubated at 37°C with 5% CO_2_. The following day, cells were infected at 37°C for 2 h with two distinct conditions: (I) 100 µL of viral samples and (II) 100 µL of viral samples with 2 µg/mL trypsin (T1426; Sigma-Aldrich). Subsequently, 1 mL of DMEM supplemented with 10% FBS and antibiotics was added to each well, and cells were incubated at 37ºC with 5% CO_2_. After 96 h, cells and supernatants (cleared by centrifugation at 2000 g for 10 min) were collected, aliquoted, and stored at -80°C. Viral RNA from the supernatants was extracted using the *Quick*-RNA Viral Kit (Zymo Research), following manufacturer’s instructions. Viral RNA from cells was extracted using phenol-chloroform. Extracted RNA was initially reverse transcribed (RT) using the SuperScript III Reverse Transcriptase (Invitrogen) and the resulting cDNA was used in a qPCR using the SYBR Green PowerUp (Applied Biosystems) and specific primers (S8 Table). Supernatants of the McAlphaCoV-Yeseras and RhBetaCoV-Murcia2022 samples collected from the initial infection were used to initiate a second passage. Supernatants from the two infection conditions (with and without trypsin treatment) were pooled and used for infecting the same cell line in which they were collected. After 3 and 6 days, cells and supernatants were collected, centrifuged at 2000 g for 10 min, aliquoted, and stored at -80ºC. Viral RNA was extracted from cells and supernatants and processed as previously described. Probe-based RT-qPCR was conducted using the TaqPath 1-Step RT-qPCR Master Mix (ThermoFisher) and specific primers/probes (S8 Table).

### Viral pseudotyping

Human codon-optimized spike constructs of the six coronaviruses initially identified were ordered as synthetic genes and cloned in a pcDNA3.1-C-HisTag vector (Genscript). For pseudotyping, T75 flasks were coated with poly-D-lysine (Gibco) for 2 h at 37°C, washed with distilled water, and seeded with 8 x 10^6^ HEK293T cells. The following day, cells were transfected with 30 µg of viral glycoprotein expression plasmid using Lipofectamine 2000 (Invitrogen) following manufacturer’s instructions. To produce negative control bald pseudotypes, cells were transfected with an empty pcDNA3.1 vector. At 24 h post-transfection, cells were inoculated at a multiplicity of infection (MOI) of 3 infectious units per cell for 1 h at 37°C with a VSV encoding GFP, lacking the glycoprotein gene G (VSVΔG-GFP), and previously pseudotyped with G. Following this, cells were washed three times with PBS and 8 mL of DMEM supplemented with 2% FBS were added. Supernatants were harvested 24 h later, cleared by centrifugation at 2000 g for 10 min, passed through a 0.45 µm filter, aliquoted, and stored at -80°C.

### Cloning of human and bat genes

The human ACE2 (hACE2)-encoding plasmid was kindly provided by Dr. Ron Geller (I2SysBio-CSIC). For each gene of interest, the sequence of the main transcript was retrieved from the NCBI RefSeq (ncbi.nlm.nih.gov/refseq), UniProt (uniprot.org/uniprotkb) or Ensembl (ensembl.org) databases, when available. The ACE2 CDS sequence from *Rhinolophus sinicus* (AGZ48803.1), *Rhinolophus cornutus* (BCG67443.1), *Myotis myotis* (XM_036305841.1) and *Pipistrellus kuhlii* (XM_036439529.2) and the APN gene sequence from *Pipistrellus kuhlii* (XM_036415540.2) were ordered as synthetic genes in a pcDNA3.1-C-FlagTag vector (GenScript). The human CDS sequences of APN (hAPN, NM_001150.3), DPP4 (hDPP4, NM_001935.4) and TMPRSS2 (hTMPRSS2, NM_005656.4) were obtained from RNA extracted using RNAzol (Sigma-Aldrich) from the CAKI-1, TK-10 and SW-620 cell lines, respectively. The ACE2 sequence (ACE2) from *Miniopterus schreibersii*, the APN sequence from *Myotis escalerai* and *Miniopterus schreibersii*, and the TMPRSS2 sequence from *Miniopterus schreibersii* and *Myotis escalerai* bat were obtained from the RNA extracted from fecal samples of the respective bat species using the NZY Total RNA Isolation kit (NZYtech). RNA was reverse transcribed into cDNA using the SuperScript IV Reverse Transcriptase (Invitrogen) and specific primers (S9 Table) following the manufacturer’s instructions. CDSs were cloned into a pcDNA3.1-C-FlagTag vector via HiFi assembly. Briefly, the pcDNA3.1-C-FlagTag vector was linearized by PCR (Forward primer: 5’-GATTACAAGGATGACGACGATAAGTG-3’; Reverse primer: 5’-GGTGGCAAGCTTAAGTTTAAACGCTAG-3’) and CDSs were amplified from cDNAs using specific primers (S9 Table) that contained a 20-nucleotide tail overlapping with the 5’ or 3’ ends of the linearized pcDNA3.1-C-Flag vector. The linearized vector and PCR-amplified sequences were purified using the DNA Clean & Concentrator-5 kit (Zymo Research), mixed in a 1:2 molar ratio, and assembled using the NEBuilder HiFi DNA Assembly Master Mix (New England Biolabs), following manufacturer’s instructions. All PCR steps were conducted using Phusion Hot Start II High-Fidelity DNA polymerase (Thermo Scientific). Assembled products were transformed into NY5α competent cells (NZYtech). Correct insertion was verified through colony PCR, using vector-specific primers (Forward primer: 5’-GAGAACCCACTGCTTACTGGC-3’; Reverse primer: 5’-AGGGTCAAGGAAGGCACG-3’) and the NZYTaq II 2x Green Master Mix (NZYtech). Plasmids were checked by whole-plasmid high-throughput sequencing (Plasmidsaurus or Eurofins). Protein expression was confirmed by western blot or pseudotype infection (see below).

### Western blot

Viral pseudotypes were pelleted by centrifugation at 30000 g for 2 h at 4°C. The pellet was lysed in 30 µL of NP-40 lysis buffer (Invitrogen) supplemented with a complete protease inhibitor (Roche) and incubated for 30 min on ice. Transfected cells were lysed in 100 µL of NP-40 lysis buffer supplemented with a complete protease inhibitor (Roche) and incubated for 30 min on ice. The lysates were then cleared by centrifugation at 15000 g for 10 min at 4°C. Viral and cellular lysates were mixed with 4x Laemmli buffer (Bio-Rad) supplemented with 10% β-mercaptoethanol and denatured at 95°C for 5 min. Proteins were separated by SDS-PAGE using pre-cast 4–20% Mini-PROTEAN TGX Gels (Bio-Rad) and transferred onto a 0.45 µm PVDF membrane (Thermo Scientific). Membranes were blocked with TBS-T (20 mM tris, 150 nM NaCl, 0.1% Tween-20, pH 7.5) supplemented with 3% bovine serum albumin (BSA; Sigma) for 1 h at room temperature (RT). Membranes were then incubated for 1 h at room temperature with the following primary antibodies: rabbit anti-HisTag (dilution 1:1000, Invitrogen PA1-983B), mouse anti-VSV-M (dilution 1:1000, clone 23H12, Kerafast EB0011), mouse anti-FlagTag (dilution 1:1000, clone M2, Sigma-Aldrich F1804) and rabbit anti-GAPDH (dilution 1:3000, Sigma-Aldrich ABS16). After three washes with TBS-T, the primary antibody was detected using a horseradish peroxidase (HRP)-conjugated anti-mouse (dilution 1:50000, Invitrogen, G-21040) or anti-rabbit (dilution 1:50000, Invitrogen, G-21234) secondary antibody. After three washes in TBS-T, signal was revealed using SuperSignal West Pico PLUS (Thermo Scientific), following manufacturer’s instructions. Images were captured using an ImageQuant LAS 500 (GE Healthcare) and analyzed using Fiji software v.2.14.0.

### Pseudotype infection assays

HEK293T cells were seeded in 96-well plates (3.5 x 10^4^ cells per well) and incubated at 37°C, 5% CO_2_ for 24 h. The following day, cells were transfected with 100 ng of plasmids encoding the indicated human and bat orthologues of ACE2, DPP4, APN and TMPRSS2, or an empty vector, using Lipofectamine 2000 (Invitrogen). After 24 h, spike-expressing pseudotypes were mixed 1:1 with a house-made anti-VSV-G monoclonal antibody and incubated for 20 min at 37°C. Cell culture medium was then removed, and cells were inoculated with 50 µL of the antibody-treated pseudotypes. After 2 h at 37°C, 50 µL of DMEM supplemented with 2% FBS was added to each well. After 24 h, plates were imaged in an Incucyte SX5 Live-Cell Analysis System (Sartorius). Cell confluence and the percentage of GFP-positive area were quantified automatically with the Incucyte Analysis software to determine the percentage of infected cells.

### Competition assays with hACE2

The soluble peptidase domain of human ACE2 (residues 19–615) was produced in a baculovirus expression system as previously described [68]. Approximately 5000 infection units of SARS-CoV-2 and RhBetaCoV-Murcia2022 spike-expressing pseudotypes were pre-incubated with serial dilutions of the soluble peptidase domain of human ACE2 or a vehicle (PBS) control for 1h at 37°C and then used to infect hACE2-expressing HEK293T cells seeded in 96-well plates. GFP-positive cells at 24 hpi were determined using the Incucyte SX5 Live-Cell Analysis System (Sartorious). The percentage of infection inhibition, a relative measure of binding affinity to ACE2, was calculated as 100× (GFP positive cells with vehicle – GFP positive cells with hACE2)/(GFP positive cells with vehicle).

## Supporting information

S1 Table Bat species assigned by visualization, number of samples, collection points and relevant data about fecal samples analized. (XLSX)

S2 Table Illumina reads and number of viral contigs obtained. (XLSX)

S3 Table Quality and identity of the viral genomes. (XLSX)

S4 Table Primers used for PCR amplification of missing regions in one partial genome. (XLSX)

S5 Table Prevalence and geographical distribution of the identified coronaviruses. (XLSX)

S6 Table Primers used to detect the identified coronaviruses by PCR. (XLSX)

S7 Table Cytochrome B bat species identification for coronavirus-positive individual samples. (XLSX)

S8 Table Primers and probes used in qPCR for the detection of coronaviruses growth in cells. (XLSX)

S9 Table Primers used for PCR amplification of bat and human sequences for HiFi cloning. (XLSX)
